# Correction: Visible-light photocatalytic performance, recovery and degradation mechanism of ternary magnetic Fe_3_O_4_/BiOBr/BiOI composite

**DOI:** 10.1039/c9ra90099c

**Published:** 2020-01-09

**Authors:** Jianhui Li, Fan Yang, Quan Zhou, Lijie Wu, Wenying Li, Ruipeng Ren, Yongkang Lv

**Affiliations:** Key Laboratory of Coal Science and Technology, Ministry of Education and Shanxi Province, Taiyuan University of Technology No. 79 Yingze West Street Taiyuan 030024 China renruipeng888@126.com

## Abstract

Correction for ‘Visible-light photocatalytic performance, recovery and degradation mechanism of ternary magnetic Fe_3_O_4_/BiOBr/BiOI composite’ by Jianhui Li *et al.*, *RSC Adv.*, 2019, **9**, 23545–23553.

The authors regret that incorrect insets were shown in Fig. 9b in the original manuscript. An SEM image of the composite before the reaction was shown in the top inset, whereas an image after the reaction should have been shown. The correct figure is shown below.

**Fig. 1:**
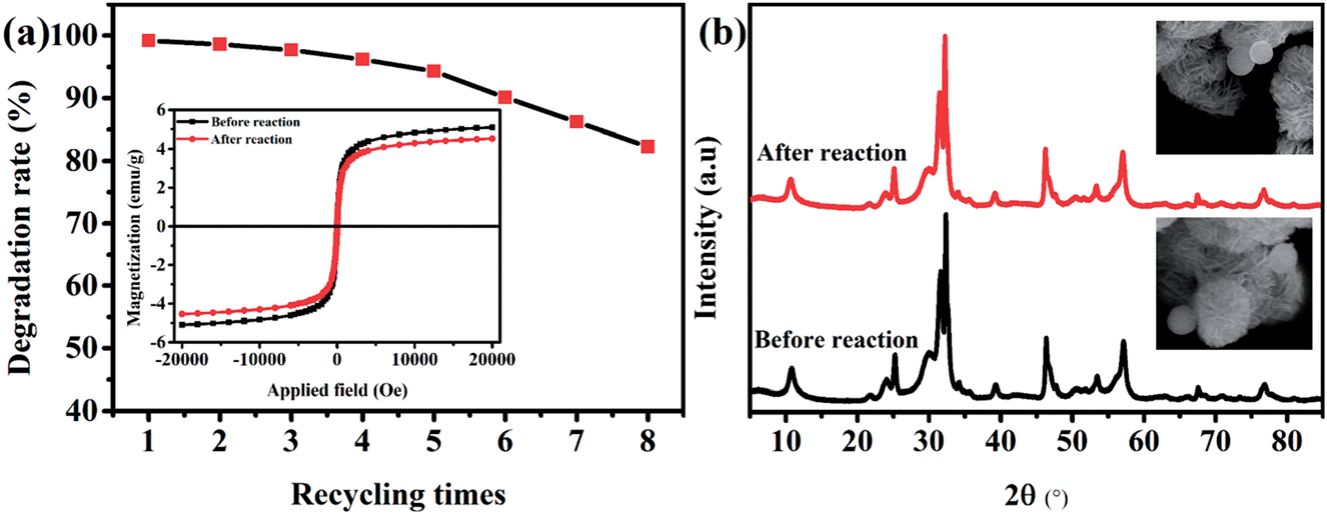
(a) Recycling utilization experiments and magnetism of Fe_3_O_4_/BiOBr/BiOI (0.4 : 3 : 1) composite before and after recycling (inset); (b) XRD patterns and SEM images (inset) of Fe_3_O_4_/BiOBr/BiOI (0.4 : 3 : 1) composite before and after recycling.

In addition, on page 23548 in the section on Morphology and composition characterization, the molar ratio of Bi : O : Br : I : Fe in the EDS analysis of the composite was incorrectly shown as 19 : 33 : 15 : 48 : 57. Therefore, the sentence beginning “What is more, the quantitative result presents the atomic…” should be corrected as shown below:

What is more, the quantitative result presents the atomic of Bi : O : Br : I : Fe in the ternary magnetic Fe_3_O_4_/BiOBr/BiOI (0.4 : 3 : 1) composite is 48 : 57 : 33 : 15 : 19, which is in line with theoretical chemometrics ratio of 20 :  28 : 15 : 5: 6 for Bi : O : Br : I : Fe in Fe_3_O_4_/BiOBr/BiOI (0.4 : 3 : 1) in view of instrumental error, implying that the mole ratio of Fe_3_O_4_, BiOBr and BiOI in the ternary magnetic Fe_3_O_4_/BiOBr/BiOI (0.4 : 3 : 1) composite is 0.4 : 3 : 1.

The Royal Society of Chemistry apologises for these errors and any consequent inconvenience to authors and readers.

## Supplementary Material

